# Membrane Surface Patterning as a Fouling Mitigation Strategy in Liquid Filtration: A Review

**DOI:** 10.3390/polym11101687

**Published:** 2019-10-15

**Authors:** Nafiu Umar Barambu, Muhammad Roil Bilad, Yusuf Wibisono, Juhana Jaafar, Teuku Meurah Indra Mahlia, Asim Laeeq Khan

**Affiliations:** 1 Chemical Engineering Department, Universiti Teknologi PETRONAS, Perak 32610, Malaysia; barambunafiu@gmail.com (N.U.B.); mroil.bilad@utp.edu.my (M.R.B.); 2 Bioprocess Engineering Program, Faculty of Agricultural Technology, Universitas Brawijaya, Malang 65141, Indonesia; 3 Advanced Membrane Technology Research Centre (AMTEC), Faculty of Chemical and Natural Resources Engineering, Universiti Teknologi Malaysia, Johor 81310, Malaysia; juhana@petroleum.utm.my; 4 School of Information, Systems and Modelling, Faculty of Engineering and Information Technology, University of Technology Sydney, Sydney, NSW 2007, Australia; TMIndra.Mahlia@uts.edu.au; 5 Department of Chemical Engineering, COMSATS University Islamabad (CUI), Lahore 54000, Pakistan; alaeeqkhan@cuilahore.edu.pk

**Keywords:** fouling mitigation, patterned membrane, operating condition, water treatment

## Abstract

Membrane fouling is seen as the main culprit that hinders the widespread of membrane application in liquid-based filtration. Therefore, fouling management is key for the successful implementation of membrane processes, and it is done across all magnitudes. For optimum operation, membrane developments and surface modifications have largely been reported, including membrane surface patterning. Membrane surface patterning involves structural modification of the membrane surface to induce secondary flow due to eddies, which mitigate foulant agglomeration and increase the effective surface area for improved permeance and antifouling properties. This paper reviews surface patterning approaches used for fouling mitigation in water and wastewater treatments. The focus is given on the pattern formation methods and their effect on overall process performances.

## 1. Introduction

Membrane technology has been used widely for many applications, such as liquid filtration, oil and gas processing, carbon capture, and many others. Many techniques of using a membrane for carbon dioxide capture and utilization have been patented [1]. In renewable energy, membrane technology has also been used for biofuel production which shows optimized end-product production by using ultrafiltration hollow fiber membranes. Biofuel production is a multimillion-dollar business, in which membrane technology can help to reduce the cost of production of biodiesel [2,3,4,5]. Membrane technology is also reliable and efficient in water and wastewater treatments and is in a state of rapid development. However, the major drawback of membrane technology is membrane fouling, which hinders its widespread application, as cleaning is costly and generates significant amounts of waste. Fouling generally refers to the blocking of pores or build-up of material on the membrane surface. Membrane fouling diminishes permeance and eventually affects the economics of the process in terms of operational and capital expenditures [6,7].

Fouling is categorized as biofouling, scaling, organic, and colloidal fouling. Biofouling involves a multi-step process where bacteria, biopolymers, and proteins adsorb onto the membrane surface or within the membrane pores. Biofouling is a major hindrance to membrane usage because unlike other types of fouling, microorganisms can grow, multiply, and relocate on the membrane surface. Scaling is a type of fouling that arises from the precipitation and deposition of salts on the membrane surface or within the membrane. Organic fouling occurs as a result of hydrocarbons coating the surface or plugging the pores of the membrane. Colloidal fouling is the accumulation of particles such as clay or silica on the surface or within the membrane [8,9,10,11,12,13,14,15,16,17,18,19,20,21,22,23]. 

Membrane fouling makes the operation more complex in accommodating for fouling management. Therefore, researchers are focusing on simplifying and inventing new strategies of fouling control. Fouling can be mitigated by controlling physical and chemical interactions between foulant materials in the feed solution and on the membrane surface [24,25].

From the perspective of membrane engineering, the surface chemistry affects the surface/foulants integration, and topography/patterning is seen as the way to alter those interactions in favor of membrane fouling control, i.e., via manipulating local mixing and hydrodynamics. Such views lead to innovative works in developing novel membrane materials having a patterned surface. However, more comprehensive applications of modifying the surface chemistry have been limited by uncertainty concerning cost, reliability, and environmental sustainability [24,25,26,27,28,29,30]. 

Membrane surface patterning is a non-chemical strategy that mitigates membrane fouling by changing the membrane or substrate surface topography. Surface patterns promote turbulence near the surface of the membrane by inducing secondary flow due to eddies which inhibit foulant accumulation and improve permeance by increasing the effective surface area [17,31,32,33,34]. Many review papers have been published regarding fouling mitigation but none on surface patterning as a fouling mitigation strategy. Therefore, this report provides a comprehensive overview of membrane surface-patterning as a fouling mitigation strategy in water and wastewater treatments.

## 2. Membrane Surface Patterning 

Surface patterning is an approach in mitigating membrane fouling by altering the membrane surface topography. The mechanism of fouling mitigation by the patterned surface is the generation of eddies induced by the patterns in combination with cross-flow velocity, which facilitates the back-diffusion of foulant to the bulk liquid [35,36,37,38,39,40].

Surface patterns reduce the membrane fouling propensity during the filtration of different feeds. Besides the hydrodynamic effect, surface patterning affects the foulant by preventing deposition of particles on the valleys if the particle size is bigger than the valley size or by altering the particle crystallization entropy when the size is about similar. Membrane surface patterning induces turbulence via local mixing near the membrane surface, requiring lower linear velocity and thus lowering the pressure loss along the module [7,41,42,43,44,45]. This advantage makes membrane surface patterning gain more attention of researchers to explore all its possible application opportunities. 

**Figure 1:**
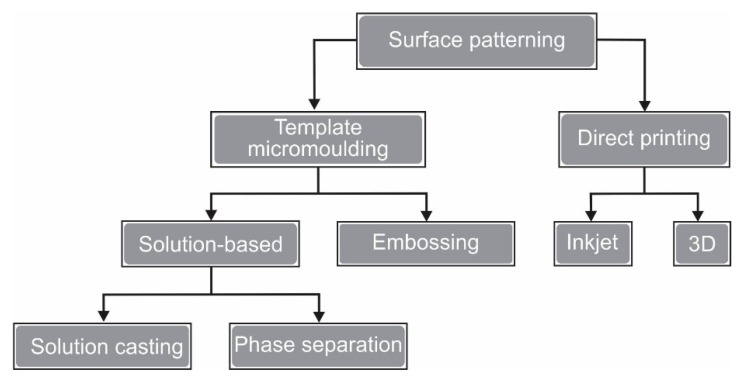
Classification surface patterning methods.

There are two main categories of surface patterning methods: template-based micromolding and direct printing [25]. Figure 1 shows the classification of surface patterning methods and the summary of those methods is depicted in Table 1.

### 2.1. Template-Based Micromolding

The template-based micromolding requires a master mold with the desired pattern. Such mold is typically fabricated directly via the lithographic process or from another master mold [32]. The negative of the master mold pattern is replicated on the membrane surface (Figure 1). There are two types of template-based micromoldings, namely solution-based and embossing micromolding.

#### 2.1.1. Solution-Based Micromolding

Solution-based micromolding employs the phase inversion process to form the solid membrane from a liquid dope solution. The dope solution is cast into a master mold, as such negative replication of the pattern in the master mold is formed in the form of polymer membrane matrix. There are two methods of solution-based micromolding, which are: solution casting and phase separation or phase inversion [25,48,49].

##### Solution Casting Micromolding

**Figure 2:**
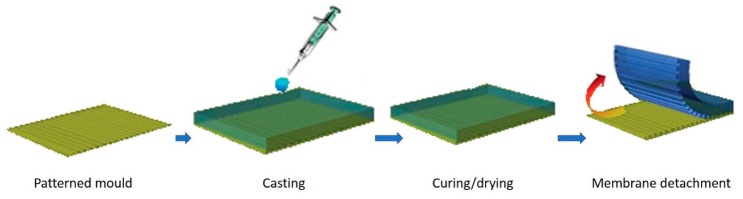
Solution casting micromolding procedure.

In solution-based micromoulding, the process of pattern formation occurs simultaneously with the membrane matrix formation. It starts with casting a liquid dope solution (i.e., Nafion) onto a master mold, and as the solvent evaporates, the solution solidifies to form the shape of the negative of the master mold. This process is relatively fast and simple which can be done even at room temperature, the patterned membrane can be developed using an elastomeric polydimethylsiloxane (PDMS) mold as illustrated in Figure 2. The patterned PDMS is used as a mold not as a stamp for the surface patterning of the Nafion film. The patterned PDMS mold is prepared using the desired thickness and shape of the patterned silicon master (the thickness of the silicon wafer itself). In a recent report, a three-dimensional (3D)-patterned membrane was fabricated from Nafion using a solvent evaporation technique. The Nafion solution is then cast onto the master mold. The solid membrane is formed after the solvent evaporation. The solid polymer matrix is then carefully peeled off the master [50,51,52,53]. 

Some parameters are important in solution casting micromolding, namely solution viscosity, solution–mold interaction, solvent evaporation rate, and adhesion of the solid polymer membrane matrix to the master mold material, which is important during the de-molding process. Nafion solution has better mechanical stability than any other polymer solution for boundary layer separation processes. This reduces the roughness experienced using solution casting micromolding techniques [54,55]. Membrane–mold adhesion during demolding affects the structure and the smoothness of the membrane surface which hinders its wide application in liquid separation processes due to the presence of a boundary layer. Membrane surface roughness facilitates accumulation of colloidal particles in the valley areas [7,43]. 

Vrijenhoek et al. demonstrated the link between surface roughness and membrane fouling in reverse osmosis and nanofiltration processes [56]. Elimelech et al. suggested that accumulation of colloidal particles happens at the valleys of the rough surfaces and as the particles accumulate, the valleys become blocked, which worsens the rate of membrane fouling [57]. 

Solution casting micromolding has been adopted in non-boundary layer processes like fuel cell membranes. In non-boundary layer separation processes, improvement of the separation surface area (permeance) is the concern of surface patterning, not the fouling [50,54,55]. Jeon et al. reported the advantages of surface-patterned Nafion membrane with a circle size of 2 μm in membrane electrode assembly. The surface-patterned membrane exhibited a 73% improvement of a referenced commercial membrane at a high power density of 1906 mW/cm^2^ when operated at 75 °C by using platinum loading of 0.4 mg/cm^2^. The remarkable improvement could be achieved thanks to the decrease in membrane electrode assembly resistance and increased surface area due to the surface pattern [58].

##### Phase Separation Micromolding

**Figure 3:**
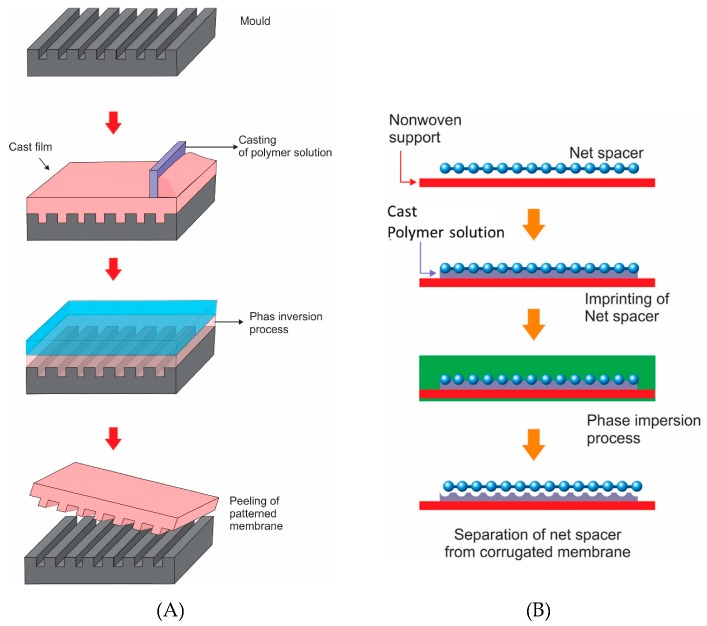
Phase separation micromolding procedures. (**A**) Membrane corrugation formed using a microstructured replica mold. (**B**) Membrane corrugation formed using net spacer.

Phase separation micromolding is identical with the solution-based micromolding. The only difference is the process of solidifying the liquid polymer solution. In solution-based micromolding, it is done by solvent evaporation, while in the phase inversion micromolding, solidification of the polymer solution is done by immersion of the cast film into a bath containing a nonsolvent, as in the traditional immersion precipitation, also known as nonsolvent induced phase separation (Figure 3A) [46,48,59]. During the evaporation of the solvent, the membrane matrix shrinks, which creates a small gap between the mold and the polymer matrices that facilitates the demolding [46,48,59,60,61]. In this method, the top surface is flat, and the pattern is formed on the bottom side, the surface in contact with the master mold. This alignment results in the formation of a dense skin layer on the patterned surface [25,48]. 

Patterns at the solvent–nonsolvent interface have also been fabricated via conventional nonsolvent vapor-induced phase separation micromolding (VI-PSmM). Unlike immersion precipitation, this process uses water from humid air as the nonsolvent to induce phase separation. After being cast onto a master mold, the cast film is exposed to a humid room. Over time, the imbibition of water that precipitates from the air destabilizes the polymer solution on top of the cast film which induces the phase inversion. The process starts from the top of the cast film gradually to the bottom and into the whole thickness of the film. Subsequently, the whole system (cast polymer solution together with the master mold) is then immersed in a nonsolvent to complete the phase inversion process in which the porous membrane matrix is fully developed [62,63,64].

Hollow fiber membranes can also be spun via VI-PSmM. It is done by using a custom-made spinneret that help to shape the outer or inner surface of the fiber. During the dry–wet spinning process, the air gap is set small. In this way, the pattern, typically on the lumen side, can be preserved by rapid coagulation in the nonsolvent [65].

There is a vast amount of advancement in phase separation micromolding processes that has been reported. A simple imprinting method was incorporated in the membrane preparation through phase inversion (Figure 3B). An improvement in permeance of 87.5% (corrugated: 15 L/m^2^·h and flat: 8 L/m^2^·h) for a membrane distillation process was achieved for a long-term operation of 50 h by a corrugated polyvinylidene difluoride that was fabricated by imprinting spacers onto the membrane surface before the phase inversion step [40]. An improvement of 50% in the effective surface area which accounts for 5–6 times the improvement in permeance was recorded for a corrugated membrane for a membrane bioreactor process via imprinting a fine and a coarse spacers onto the cast film before the phase inversion step [66].

#### 2.1.2. Embossing Micromolding

**Figure 4:**
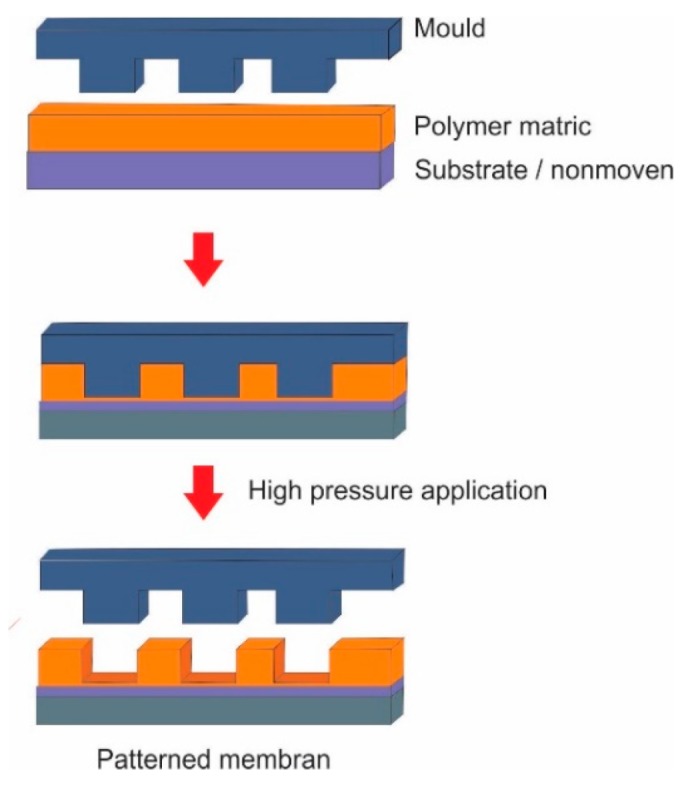
Illustration of embossing micromolding procedure.

Embossing micromolding (EM) techniques involve stamping of a rigid patterned master mold under high pressure (wafer-based) or high temperature and pressure (thermal embossing or nanoimprinting) on the polymer surface to replicate the patterns of the master mold (Figure 4). EM can be done using thermal, nanoimprinting, and wafer-based embossing. Careful control of the processing parameters (*T*, *P*, *t*) is the key to achieving high fidelity of the pattern in EM. Thermal embossing micromolding (TEM) uses high temperatures above the glass transition temperature *T*_g_ of the polymer and a pressure in range of 20–100 bar to “imprint” the topographic features onto a flat polymeric membrane. However, TEM was modified (nanoimprinting lithography) by using temperatures just below the polymer glass transition temperature due to pore sealing and rupture observed by earlier approaches [24]. In nanoimprinting, the temperature is slightly lower or just below the *T*_g_, and a pressure of 20–100 bar is beneficial in avoiding pore sealing and defects that occur in the TEM. The pore sealing in TEM was attributed to the viscous flow of the polymer at temperatures above the glass transition of the polymer [24,32,67]. 

Wafer-based embossing, an improved version of the thermal and nanoimprinting processes, creates the patterned surface at room temperature but employs a higher pressure of >200 bar for embossing. This modification is considered an improvement from the traditional TEM complexity by eliminating heating and cooling steps, which also offers economic competitiveness [25].

### 2.2. Direct Printing

Direct printing is categorized into ink-jet printing and 3D printing, which are detailed below.

#### 2.2.1. Inkjet Printing

**Figure 5:**
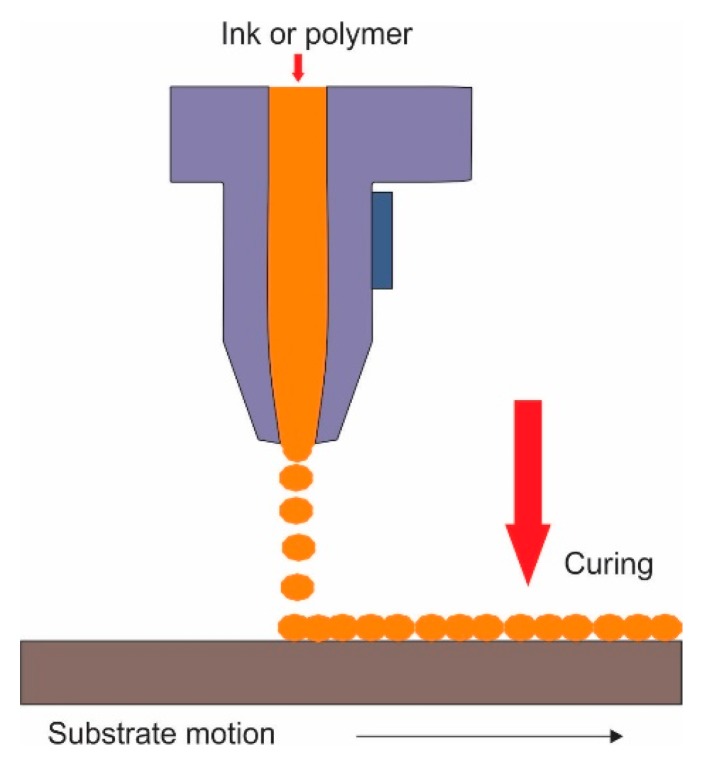
Basic illustration of direct printing procedure.

Inkjet printing is a technique that involves solvent evaporation of structured droplets of a solution to form computer-based designed objects (Figure 5) [28]. It has been used exclusively for the chemical formation of surface patterns on polymers. Therefore, it is briefly discussed as it is beyond the scope of this review. Bandalov et al. incorporated inkjet printing with interfacial polymerization for the fabrication of thin film composite membrane for water desalination process. The membrane showed ~97.2% salt rejection and a ~26.4% increase in permeance [68]. Gao et al. generated a patterned structure layer-by-layer on a microfiltration membrane with 0.2 µm pore diameter by incorporating inkjet printing with template synthesis [69].

#### 2.2.2. Additive Manufacturing (3D Printing)

Three-dimensional printing, also known as microstereolithography and rapid prototyping, is the construction of 3D objects based on computer-designed models. This technique allows the formation of geometrically complex shapes and features via the layer-by-layer deposition of polymeric materials (Figure 5) [70]. Three-dimensional printing has revolutionized the traditional prototyping and manufacturing industry that depends on the expensive and time-consuming conventional methods. In 3D printing, the design and fabrication of micro- and macro-structure membranes can be controlled in one go. Three-dimensional printing is categorized into: photopolymerization, powder, material extrusion, and lamination [47,70,71,72,73,74]. There is much ongoing research on the advancement of continuous liquid interface production (CLIP), the ability for 3D printers to accommodate to more polymers, the reduction in processing time, and improvement in resolution.

Photopolymerization is popular for fabricating polymeric membrane for liquid-based filtration. It is not only economically attractive, but also offers a high resolution. In photopolymerization, photo-reactive polymers (photopolymers) are cured with the help of a lasers. For example, in laser-lithography also known as stereolithography (SLA), an ultraviolet laser is employed for tracing and curing a model’s cross-section. The formed trace is then coated with a resin layer. The process is repeated until the entire structure is formed until finally the object is cured in an ultraviolet oven [75]. 

Direct light processing (DLP) improved traditional 3D printing by using normal light instead of laser. CLIP techniques based on DLP are a technological breakthrough in photopolymerization, where printing times can be reduced by 25 to 100 times. DLP techniques require a mechanical separation of the cured layer from the bottom of the resin vat, followed by resin re-coating before the next layer is exposed. CLIP forms an oxygen-containing “dead zone” to reduce mechanical movement. The presence of the dead zone inhibits adhesion to the resin vat for multi-layer printing which then eliminates the separation step in the traditional SLA printers and radically reduces the construction time while still offering high resolution [70,74,75,76].

Al-Shimmery et al. fabricated a 3D-patterned polyethersulfone membrane. It was done by casting the polymer atop an acrylonitrile butadiene styrene-like 3D-printed substrate. The patterned membrane offered 30% higher pure water permeance compared to a flat membrane. The membrane was tested for filtration of feed comprised of oil-in-water emulsions. When treating oil/water emulsions, the patterned membrane had a 52% higher permeance recovery ratio after the first filtration cycle, without reducing the rejection rate. It maintained constant hydraulic performance after five filtration cycles without requiring chemical cleaning [77]. Such performance was much better than that of the flat membrane, in which it severely fouled even after the first cycle.

Mazinani et al. fabricated 3D-printed membrane by physically attached a polymeric membrane on top of 3D-patterned support. The membrane active layer was prepared from polyethersulfone polymer. The attachment of the polymeric membrane matrix was done through vacuum pressure. The performance of the resulting membranes was analyzed through a crossflow filtration set-up using bovine serum albumin (BSA) as a feed. The results showed a 10% improvement in pure water permeance (PWP) of the patterned membrane against the flat membrane. The patterned membrane maintained a 87% permeance recovery ratio even after the 10th filtration cycle [78].

## 3. Operation Conditions of Patterned Membranes 

**Figure 6:**
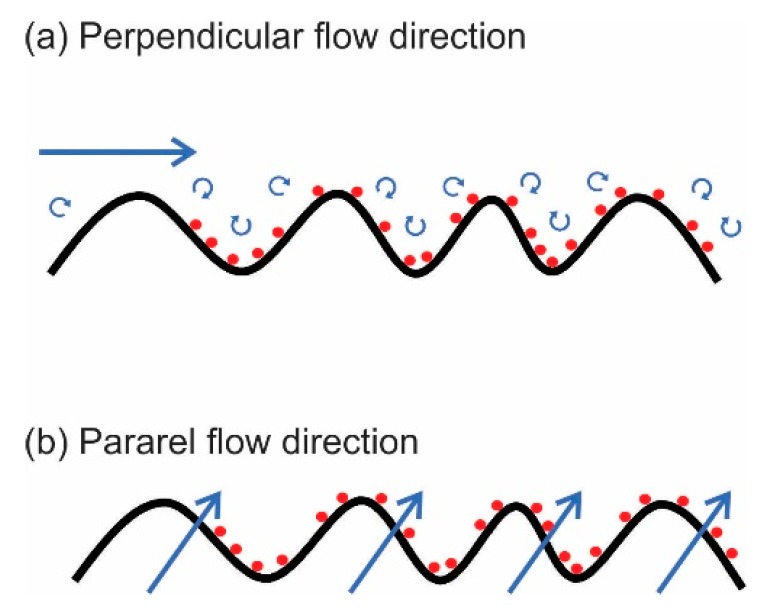
Illustration on the effect of flow orientation on membrane fouling propensity under (**a**) perpendicular flow direction and (**b**) under parallel flow direction.

The objectives of surface patterning can only be achieved by operating at an appropriate flow angle (Figure 6). A 0° flow angle (flow parallel to patterns) shows significant fouling, whereas a 45° or 90° flow angle (flow perpendicular to patterns) shows a significant reduction in fouling [79]. This reduction in fouling is caused by eddies that are formed at the membrane surface, inducing localized mixing such that foulant materials are less likely to come in contact with and adhere to the membrane [80]. 

The influence of the membrane patterns is more pronounced under higher feed crossflow velocities. Higher corrugation angles increase membrane permeance reaching the highest value at 90°, induce maximum local mixing. However, surface patterning incurs higher pressure drops along the membrane module than the flat surface owing to flow resistance and friction exhibited by the surface patterns to the axial flow [17,41,80]. Therefore, operating at higher feed flow velocity for patterned membrane entails a significant increase in energy consumption for pumping countering the benefit of higher hydraulic productivity. Scott et al. demonstrated an increase in energy savings of 88% for the patterned membrane as compared to a flat membrane of the same area by calculating the power ratio of the two membranes [39]. The summary of recent reports on performance enhancements of filtration due to surface patterning is shown in Table 2. 

## 4. Importance of Surface Patterning for Various Membrane Processes

Membrane fouling is an established problem in most liquid-based filtration processes [6,87]. Membrane surface patterning has been explored as a viable alternative for fouling control due to economical and sustainability considerations. Apart from inducing turbulence flow of the feed, surface patterning also increases the effective surface area for improved permeability [17,41]. The 3D pattern induces turbulence flow by promoting local mixing and hence inhibits accumulation of foulant on the membrane surface [26,66,81,82,88].

Membrane surface patterning was applied using phase separation micro-molding for a thin film composite (TFC) used for reverse osmosis processes. Elsherbiny et al. found a ~210%–240% improvement in water permeability for the patterned membrane as compared to a flat TFC membrane without sacrificing the membrane selectivity [31]. Maruf et al. imposed a pattern on a TFC membrane using interfacial polymerization by utilizing patterned ultrafiltration as a support and reported a 22% improvement in permeability [86].

Kharraz et al. applied membrane surface patterning for fouling control in membrane bioreactor. They reported a 50% improvement in the membrane effective surface area, contributing to a 5–6-fold higher permeance than a flat reference membrane [66]. Kim et al. patterned a hollow-fiber membrane for membrane bioreactor application and found a 25% improvement in water flux of the patterned membrane against the flat membrane [81]. Similarly, ribbed membrane also provide advantages in membrane bioreactor using a novel silica membrane material [45].

Choi et al. patterned ultrafiltration membranes and evaluated the performance of the patterned and flat membranes by subjecting them to a crossflow filtration set-up with 1.1 µm polystyrene latex beads as feed. Improvement in water flux of 38.6% of the patterned membrane against the flat one was observed. The mass of particle deposition on the patterned membrane surface was found to be 10 µg/cm^2^, corresponding to one-third of the flat membrane [44]. 

Surface patterning has been reported to improve the performance of the membrane distillation process. Xie et al. applied membrane surface patterning in a membrane distillation process. The patterned membrane maintained a steady-state water flux of almost 25 L.m^−2^·h^−1^, 67% higher than a reference flat membrane. The BSA rejection was found to be a 4.2-fold improvement for the patterned membrane as compared to the pristine membrane [88]. Nawi et al. reported an 87.5% improvement in permeance for a corrugated membrane against the pristine in a prolonged operation of 50 h in a membrane distillation process [40].

Izak et al. patterned, in an organophilic pervaporation process, a membrane for hexyl acetate recovery from C_4_mim-BF_4_. The result showed a 14% improvement in hexyl acetate recovery against the flat membrane tested under the same conditions [89]. Overall, reports show conclusive results on the positive impact of surface patterning in enhancing membrane processes′ hydraulic performance (i.e., pressure-driven, temperature-driven, and pervaporation performance) without altering the rejection performance.

## 5. Conclusions and Perspectives

Membrane surface patterning has the potential to mitigate fouling and improve energy saving by inducing turbulence near the membrane surface, improving the effective surface are and preventing liquid wetting (membrane distillation) and crystallization (desalination) as discussed in this review. However, there are challenges remaining for the implementation of available surface patterning techniques. The EM process has the limitations of shape designs, pore deformation, low fidelity, and high energy consumption. PSmM has the limitations of low speed, low fidelity, and shape designs. Three-dimensional printing has the limitations of low resolution, low speed, and limited polymers adoption. Researchers are focusing on improvement of PSmM to fabricate complex designs, high fidelity, and speed; of EM pore deformation, the construction of complex designs, high fidelity, and energy consumption; and of 3D resolution, speed, and adoption of all polymers.

## Figures and Tables

**Table 1:** Summary of membrane surface patterning methods.

Description of Patterning Method	Advantage	Disadvantage	Ref.
EM is a stamping of a rigid patterned master mold under high pressures and temperatures and pressure on the polymer surface to replicate a negative of the master mold patterns	Good resolution High speed	High energy consumption Simple shapes only Low fidelity	[33]
PSmM involves phase inversion to shape the pattern from a liquid dope solution which precipitates into the solid phase on the pattern features of a master mold.	Good resolution Easy to control	Slow Simple shapes only Low fidelity	[46]
3D printing constructs the pattern structure through layer-by-layer deposition according to the input 3D model.	Free of geometry Easy to control High fidelity	Limited polymers Moderate resolution	[47]
Inkjet printing deposits the droplet of solution jet to form a 3D pattern solidified due to solvent evaporation	Easy to control High fidelity	Limited application Moderate resolution	[28]
SCmM casts a Nafion polymer solution onto a master mold, and when it solidifies, the solid pattern is formed as the negative of the master mold.	Easy to control Good resolution	Poor fidelity Limited application	[48]

EM: embossing micromolding, PSmM: phase separation micromolding, SCmM: solution casting micromolding, 3D: three-dimensional.

**Table 2:** Recent reports on performance of patterned membrane for water treatment.

Patterning Technique	Feed	Major Findings	Ref.
PSmM	Activated sludge	20–25% improvement in permeance flux and 3 times as fouling resistant	[81]
PSmM	2000 ppm NaCl solution	210% improvement in permeability	[31]
PSmM	2 µm diameter latex bead suspensions	5.1-fold improvement in mass of particle deposition on membrane surface	[17]
PSmM	Activated sludge	Permeance: 5804 L/m^2^·h.bar (fine), 4241 L/m^2^·h.bar (coarse) and 943 L/m^2^·h.bar (flat)	[66]
VI-PSmM	Activated sludge	~20% permeance improvement	[82]
VI-PSmM	Yeast suspensions	103% improvement in surface area	[83]
3D printing	BSA	Reduced normalized flux: 19–24% with parallel stripes, 13% with flat (no pattern) and 5% with perpendicular stripes	[84]
3D printing	BSA	Wavy membrane has 10% better PWP than flat membrane. Wavy membrane has 87% PRR while flat has 53%	[78]
3D printing	oil-in-water emulsion 0.3–0.5 vol %	Wavy membrane has 30% better PWP than flat membrane	[77]
Inkjet printing	Saline water	~26.4% increase in permeance and ~97.2% salt rejection	[68]
TEM	BSA	104% increase in flux recovery ratio ~91% in permeance	[85]
NIL	2000 ppm NaCl solution	240% improvement in permeability	[31]
NIL	1 g/L NaCl solution	22% improvement in permeability at 0.01 wt % MDP concentration	[86]

PSmM: Phase separation micromolding, VI-PSmM: Vapor induced phase separation micromolding, 3D printing: Three-dimensional printing, TEM: Thermal embossing micromolding, BSA: Bovine serum albumin, PWP: Pure water permeance, PRR: Permeance recovery ratio, NIL: Nanoimprinting lithography.

## Abbreviations

3D	Three-dimensional
BSA	Bovine serum albumin
CLIP	Continuous liquid interface production
DLP	Direct light processing
EM	Embossing micromolding
NIL	Nanoimprinting lithography
PDMS	Polydimethylsiloxane
PES	Polyethersulfone
PRR	Permeance recovery ratio
PSmM	Phase separation micromolding
PWP	Pure water permeance
SCmM	Solution casting micromolding
SLA	Stereolithography
TEM	Thermal embossing micromolding
TFC	Thin film composite
VI-PSmM	Vapor-induced phase separation micromolding
